# Supplementary material for: TGFβ blocks IFNα/β release and tumor rejection in spontaneous mammary tumors
Source: Nat Commun. 2019 Sep 11;10:4131. doi: 10.1038/s41467-019-11998-w (PMC6739328; doi:10.1038/s41467-019-11998-w)
Supplement: Supplementary file 1 — Supplementary Information [file 41467_2019_11998_MOESM1_ESM.pdf]

Supplementary Information

TGFβ blocks IFNα/β release and tumor rejection in spontaneous mammary tumors

Guerin et al.

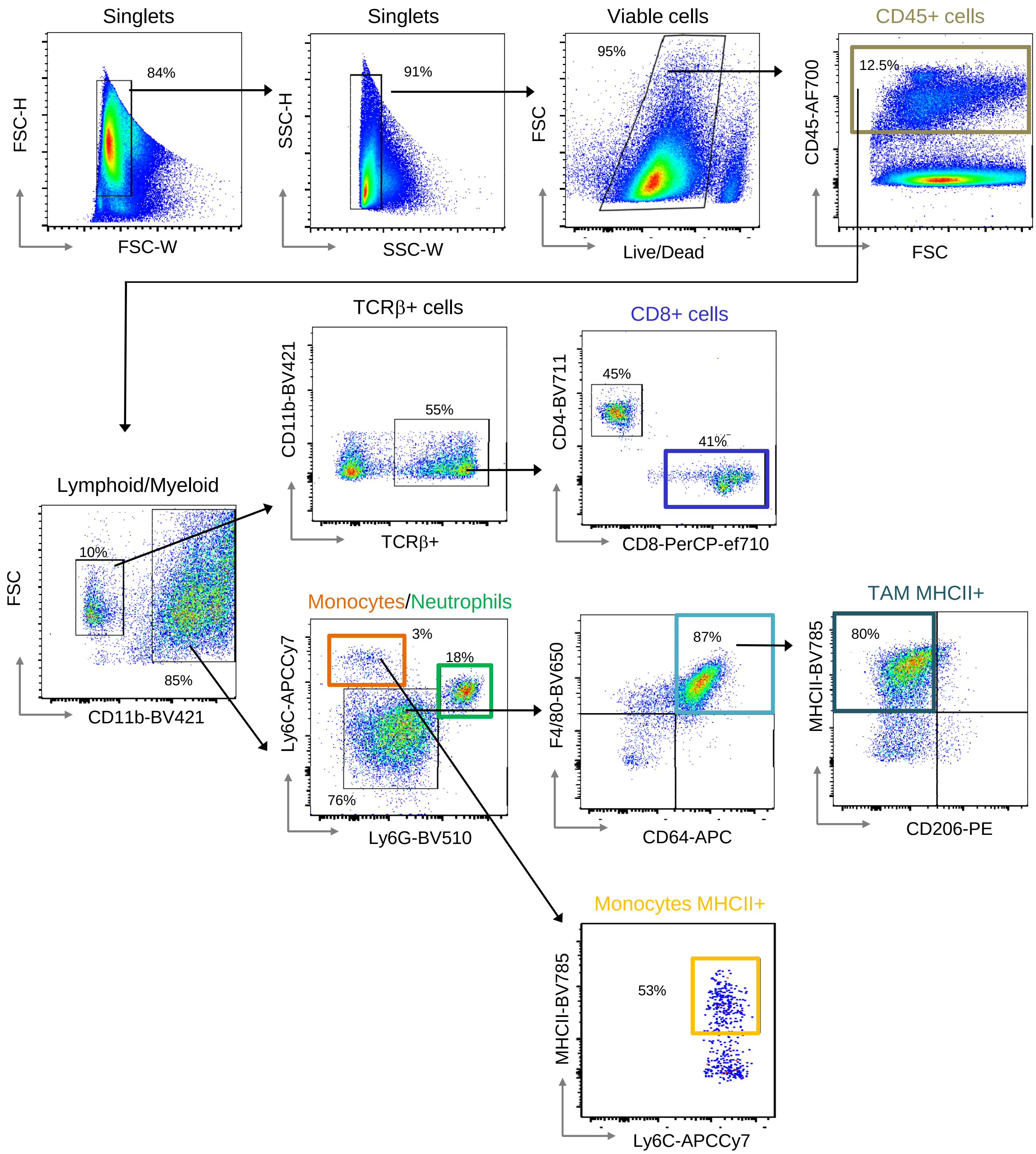

Supplementary Figure 1. FACS sequential gating strategies.

Tumor Immune infiltrating cells were identified after exclusion of doublets and dead cells (Live/dead^{neg};CD45^{+}). Sequential gating allows for identification of CD8 T cells (Live/dead^{neg};CD45^{+};CD11b^{-};TCRβ^{+};CD8^{+}), monocytes (Live/dead^{neg};CD45^{+};CD11b^{+};Ly6C^{+}/Ly6G^{-}), neutrophils (Live/dead^{neg};CD45^{+};CD11b^{+};Ly6C^{lo}/Ly6G^{+}), TAM (Live/dead^{neg};CD45^{+};CD11b^{+};Ly6C^{-}/Ly6G^{-}; CD64^{+}/F480^{+}), including MHC II^{+} TAM subset.

a

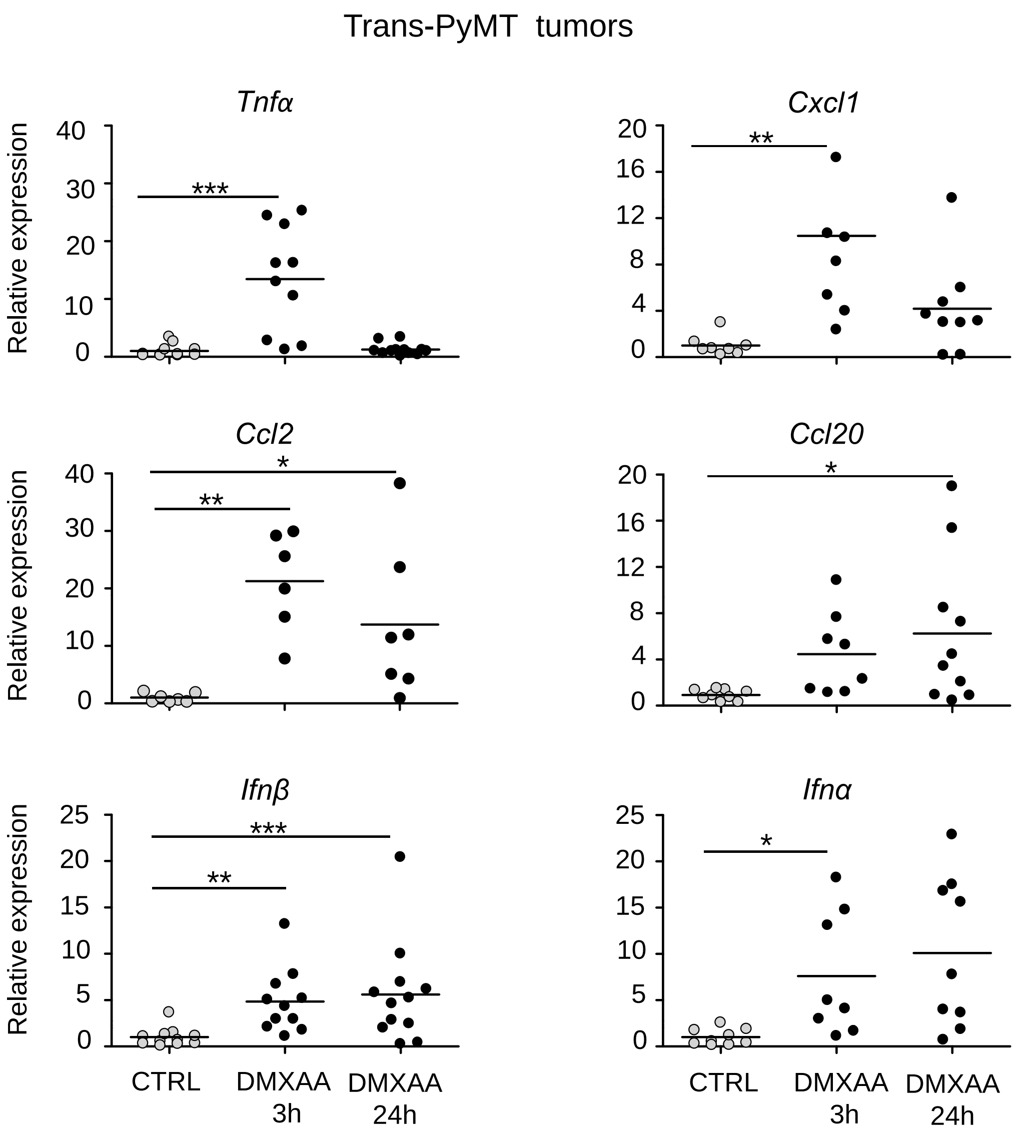
Trans-PyMT tumors
Tnfα
Cxcl1
Ccl2
Ccl20
Ifnβ
Ifnα
Relative expression
40
30
20
10
0
16
12
8
4
25
15
5
***
**
*
CTRL
DMXAA 3h
DMXAA 24h

b

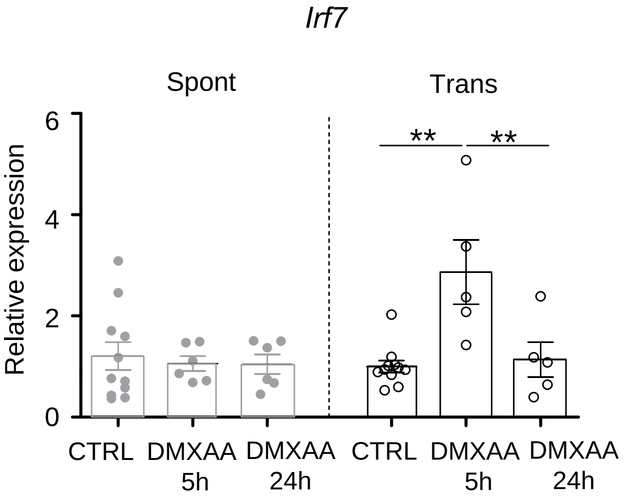
Irf7
Spont
Trans
6
4
2
0
Relative expression
**
**
CTRL
DMXAA 5h
DMXAA 24h
CTRL
DMXAA 5h
DMXAA 24h

Supplementary Figure 2. Cytokines and chemokines expression induced by DMXAA in Trans-PyMT tumors. **(a)** Trans-PyMT mice were injected i.p. with DMXAA (black circles) or DMSO (grey circles), and mRNA levels of cytokines and chemokines were measured after 3 hours or 24 hours as indicated in Fig. 2a. The relative expression, normalized to Trans-PyMT control tumors, is shown and the median is indicated. Cumulative data, with the Median, are shown. Data of 7-11 mice, from 3-5 independent experiments are shown. Tukey's Multiple Comparison Test. * $p<0.05$; ** $p<0.01$; *** $p<0.001$. **(b)** Spont and Trans-PyMT mice were injected i.p. with DMXAA or DMSO, and mRNA levels of cytokines and chemokines were measured after 5 hours or 24 hours. The relative expression, normalized to Trans-PyMT control tumors, is shown. Results are expressed as mean ± SEM. Data were obtained from Trans-PyMT mice: *n*=9 CTRL and *n*=5 DMXAA treated and from Spont-PyMT mice: *n*=5 CTRL and *n*=3 DMXAA treated, from 3 independent experiments. Tukey's Multiple Comparison Test. * $p<0.05$; ** $p<0.01$; *** $p<0.001$. Source Data are provided as a Source Data file.

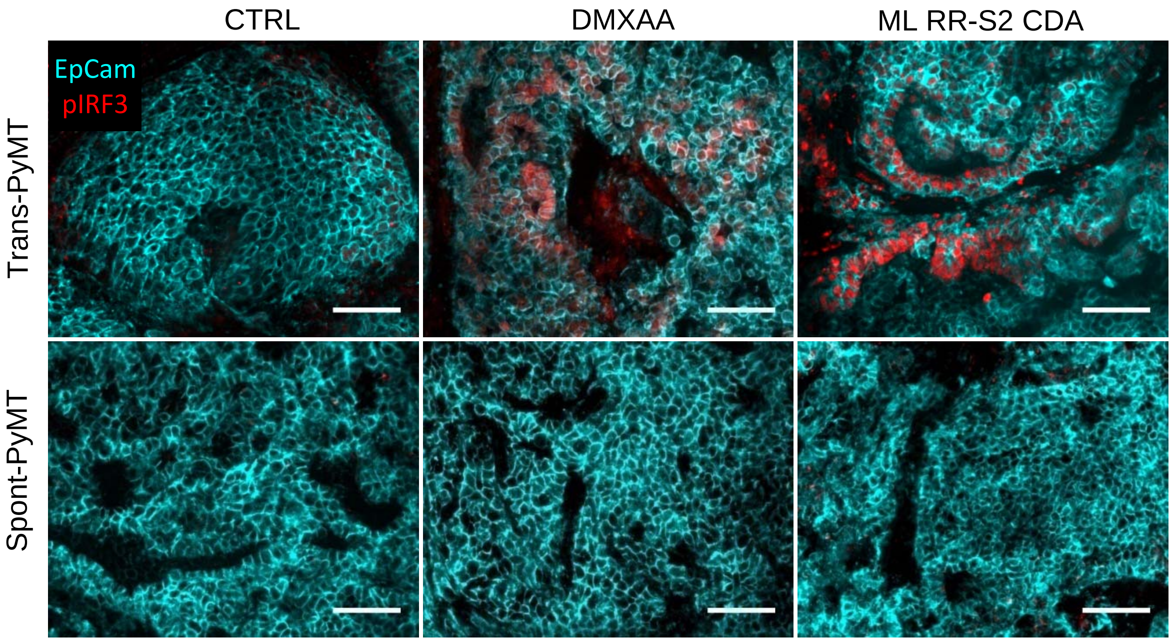

Supplementary Figure 3. I.t. injection of DMXAA or ML RR-S2 CDA induced pIRF3 in Trans-PyMT, but not in Spont-PyMT mice. Trans- and Spont-PyMT mice were injected i.t. with DMXAA (1x 500µg) or ML RR-S2 CDA (1x 50µg). Tumors were collected 3 hours later and tumor slices stained with anti-EpCAM (blue) and anti-pIRF3 (red) mAbs. Scale bars = 50 µm. Images representative of Spont-PyMT mice: *n*=3 (CTRL), n=2 (DMXAA), *n*=2 (ML RR-S2 CDA); Trans-PyMT mice: *n*=2 (CTRL), *n*=2 (DMXAA), *n*=2 (ML RR-S2 CDA), from 5 independent experiments, are shown.

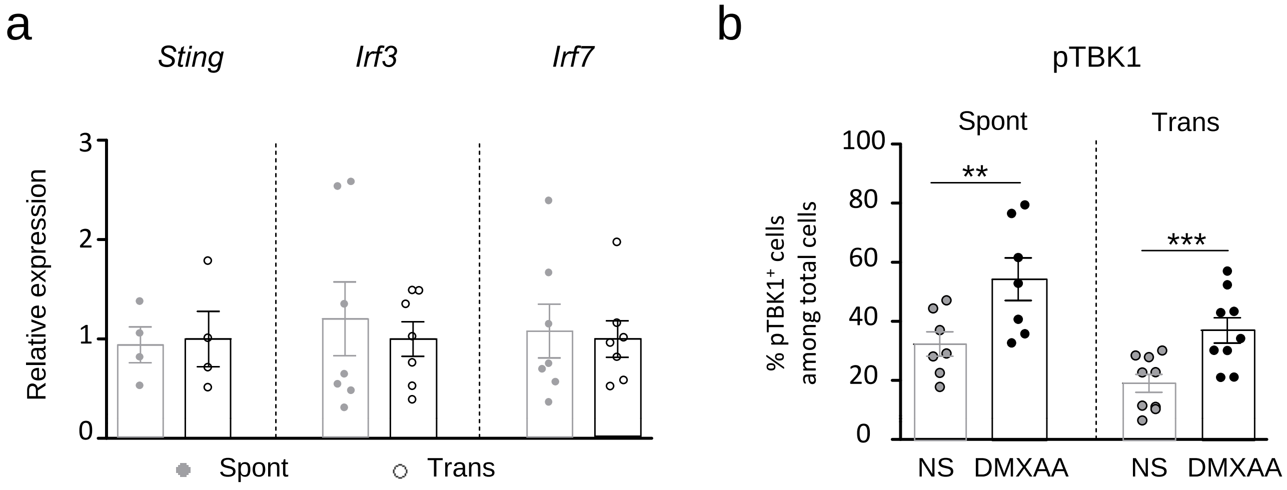

Supplementary Figure 4. Induction of *Sting, Irf3, Irf7* expression and activation of TBK1 after DMXAA

(a) Expression of *Sting*, *Irf3* and *Irf7* mRNA transcripts in tumors of Spont-PyMT mice (grey circles) and Trans-PyMT (open circles) mice. The relative expression is shown, normalized to Trans-PyMT tumors. Spont-PyMT mice: *n*=3, Trans-PyMT mice: *n*=4 (*Sting*) or 7 (*Irf3* and *Irf7*) from 3 independent experiments. **(b)** Similar activation of TBK1 in Spont- and Trans-PyMT tumors after DMXAA stimulation in vitro. Tumor cell suspensions from Spont-PyMT and Trans-PyMT mice were prepared as in Fig. 5b, cultured for 3 hours in vitro with DMXAA (250 µg/ml) or left untreated, then the levels of TBK1 activation (pTBK1) was determined by flow cytometry. The percentage of pTBK1^{+} cells in DMXAA-treated versus control-treated conditions is shown. Spont-PyMT mice: *n*=3, Trans-PyMT mice: *n*=8. Data are from 3 independent experiments. Results are expressed as mean ± SEM. Paired *t*-Test. * p<0.05; ** p<0.01; *** p<0.001. Source Data are provided as a Source Data file.

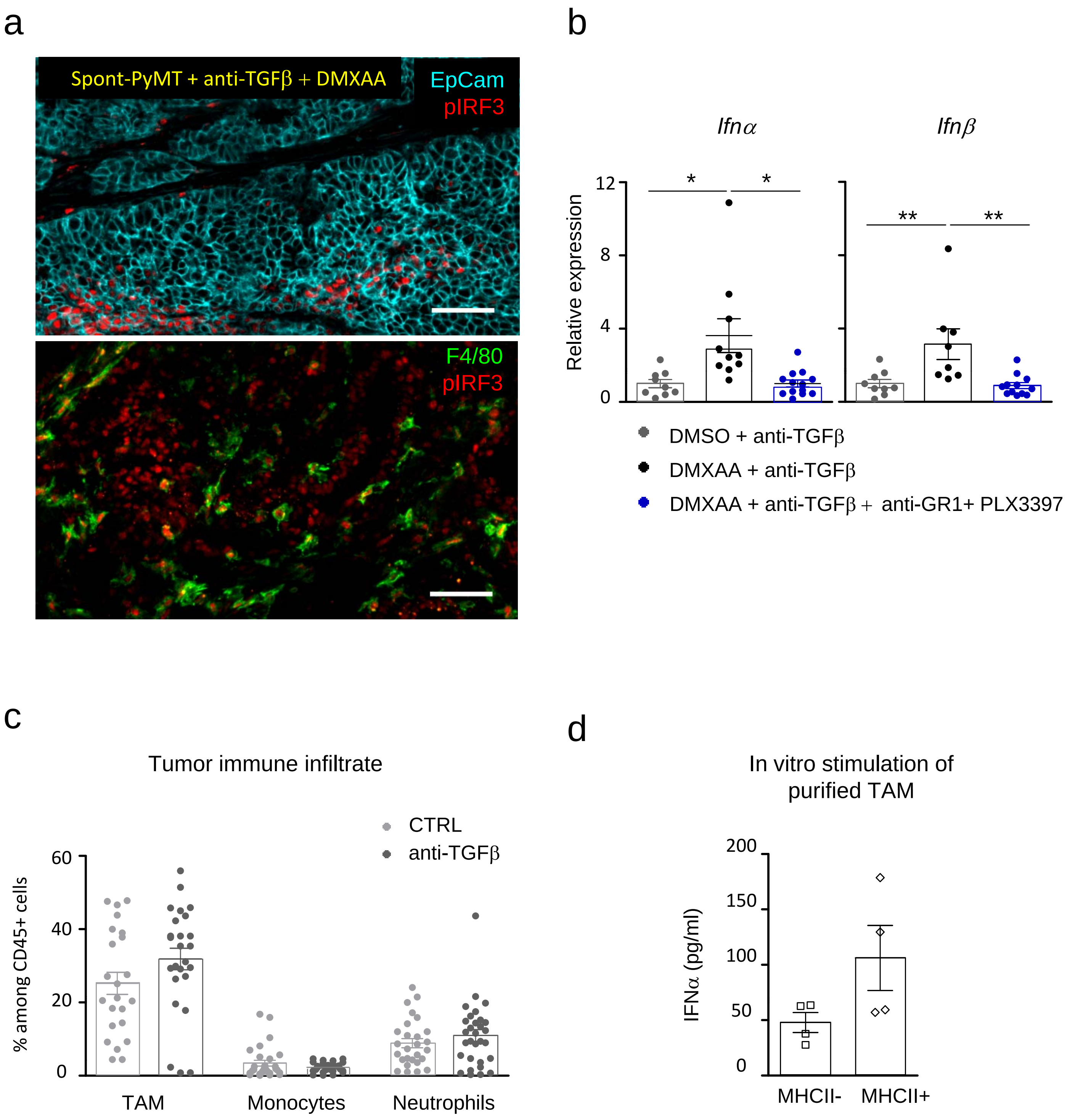

a
Spont-PyMT + anti-TGFβ + DMXAA
EpCam
pIRF3
F4/80
pIRF3
b
Ifnα
Ifnβ
Relative expression
12
8
4
0
*
*
**
**
DMSO + anti-TGFβ
DMXAA + anti-TGFβ
DMXAA + anti-TGFβ + anti-GR1+ PLX3397
c
Tumor immune infiltrate
CTRL
anti-TGFβ
% among CD45+ cells
60
40
20
0
TAM
Monocytes
Neutrophils
d
In vitro stimulation of
purified TAM
IFNα (pg/ml)
200
150
100
50
0
MHCII-
MHCII+

Supplementary Figure 5. Blocking TGFβ allows DMXAA-induced pIRF3 and IFNα production in TAM.

(a) Spont-PyMT mice, treated with an anti-TGFβ, received one i.p. injection of DMXAA. Tumors were stained as in Fig. 5a. Two different image fields show that pIRF3$^+$ cells may be tumor cells (EpCAM$^+$, blue, top), or myeloid cells (F4/80$^+$, green, bottom). Scale bars 50 µm. Images representative of 4 treated Spont-PyMT mice. **(b)** Spont-PyMT mice treated with an anti-TGFβ alone or in combination with anti-GR1 (day-4 and day-1) and PLX3397 (from day-2 to day +1), received DMXAA or DMSO on day0. *Ifnα* and *Ifnβ* gene expression were measured in tumors on day 1. The relative expression is shown, normalized to control tumors (DMSO, anti-TGFβ treated mice). Spont-PyMT mice treated with: DMSO + anti-TGFβ (*n*=3) (grey circles), DMXAA + anti-TGFβ (*n*=3) (black circles), DMXAA + anti-TGFβ + anti-GR1 and PLX3397 (*n*=4) (blue circles) from 4 independent experiments. Tukey's Multiple Comparison Test. * $p<0.05$; ** $p<0.01$; *** $p<0.001$. **(c)** The proportion of TAM, monocytes and neutrophils among CD45$^+$ cells in tumors was determined in Spont-PyMT mice pre-treated (dark grey circles) or not (light grey circles) with an anti-TGFβ. Spont-PyMT mice: n= 3, anti-TGFβ Spont-PyMT mice: *n*=3 from 3 independent experiments. **(d)** MHC II$^+$ (open diamonds) and MHC II$^-$ (open squares) TAM from Spont-PyMT tumors were sorted, stimulated overnight with DMXAA, and IFNα released was quantified by ELISA. *n*=4 mice from 4 independent experiments. Results are expressed as mean ± SEM. Source Data are provided as a Source Data file.

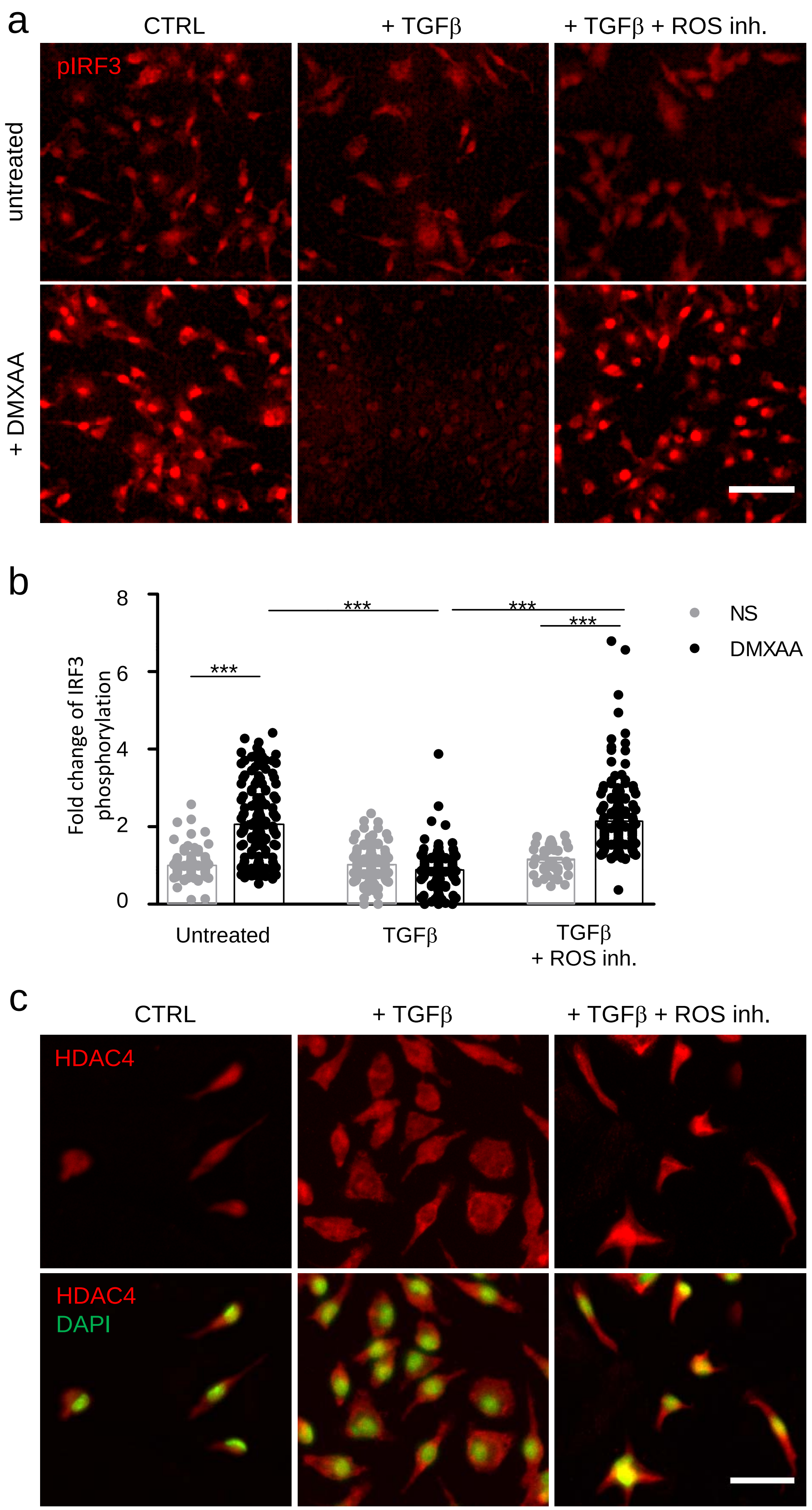
a
CTRL
+ TGFβ
+ TGFβ + ROS inh.
untreated
+ DMXAA
pIRF3
b
Fold change of IRF3 phosphorylation
8
6
4
2
0
***
***
***
***
NS
DMXAA
Untreated
TGFβ
TGFβ + ROS inh.
c
CTRL
+ TGFβ
+ TGFβ + ROS inh.
HDAC4
HDAC4
DAPI

Supplementary Figure 6. TGFβ alters pIRF3 in DMXAA-stimulated BMDM by the nuclear export of HDAC4.

(a) BMDM were cultured overnight with TGFβ (5ng/ml), TGFβ + ROS inhibitor (1nM) or left untreated. Cells were then stimulated or not with DMXAA (250µg/ml) for 3 hours, then stained with anti-pIRF3 Ab (red). Scale bar = 50 µm. **(b)** The increase in the surface covered by pIRF3^{+} staining was measured in DMXAA-stimulated (black circles) compared to unstimulated (grey circles) cells. Results are expressed as mean ± SEM from the cumulative data of 3 independent experiments. Tukey's Multiple Comparison Test . * $p<0.05$; ** $p<0.01$; *** $p<0.001$. **(c)** BMDM exposed to TGFβ or TGFβ + ROS inhibitor as in **a**, were stained with anti-HDAC4 Ab (red) and, for the nucleus, DAPI (green). Top: HDAC4; Bottom: overlay with DAPI. Scale bar = 30 µm. Images from one experiment are shown and are representative of 3 independent experiments. Source Data are provided as a Source Data file.

Ifnα4	TGATGAGCTACTACTGGTCAGC	GATCTCTTAGCACAAGGATGGC
Ifnβ	TGGGTGGAATGAGACTATTGTTG	CTCCCACGTCAATCTTTCTCT
Ccl2	CATCCACGTGTTGGCTCA	GATCATCTTGCTGGTGAATGAGT
Ccl20	GTGGGTTTCACAAGACAGATG	TTTTCACCCAGTTCTGCTTTG
Cxcl1	GCTGGGATTCACCTCAATG	TGGGGACACCTTTTAGCATC
Tnfα	AATGGCCTCCCTCTCATCAGTT	CGAATTTTGAGAAGATGATCTGAGTGT
Tgfβ	TGACGTCACTGGAGTTGTACGG	GGTTCATGTCATGGATGGTGC
Gapdh	TGTGTCCGTCGTGGATCTGA	TTGCTGTTGAAGTCGCAG

Supplementary Table 1. Primer sequences for qPCR

	DMSO	DMSO + anti-TGFβ	DMXAA	DMXAA + anti-TGFβ	DMXAA + anti-TGFβ + anti-IFNαR	DMXAA + anti-TGFβ + anti-CD8
DMSO	1					
DMSO + anti-TGFβ	0.8668013	1				
DMXAA	0.0790010	0.1608655	1			
DMXAA + anti-TGFβ	0.000003	0.0003630	0.00009	1		
DMXAA + anti-TGFβ + anti-IFNαR	0.4167757	0.3180283	0.0486888	0.0003630	1	
DMXAA + anti-TGFβ + anti-CD8	0.0001876	0.0023256	0.0018427	0.7794144	0.0023256	1

Supplementary Table 2. p values related to *in vivo* treatments shown in Fig. 6d. P values were obtained after running a Kolmogorov-Smirnov test on the distribution of tumor growths across mice.
