# Supplementary material for: TGFβ blocks IFNα/β release and tumor rejection in spontaneous mammary tumors
Source: Nat Commun. 2019 Sep 11;10:4131. doi: 10.1038/s41467-019-11998-w (PMC6739328; doi:10.1038/s41467-019-11998-w)
Supplement: Supplementary file 3 — Reporting Summary [file 41467_2019_11998_MOESM3_ESM.pdf]

nature research

Corresponding author(s): Nadège Bercovici
Last updated by author(s): Aug 7, 2019

Reporting Summary

Nature Research wishes to improve the reproducibility of the work that we publish. This form provides structure for consistency and transparency in reporting. For further information on Nature Research policies, see Authors & Referees and the Editorial Policy Checklist.

Statistics

For all statistical analyses, confirm that the following items are present in the figure legend, table legend, main text, or Methods section.

n/a	Confirmed	
☐	☒	The exact sample size (*n*) for each experimental group/condition, given as a discrete number and unit of measurement
☐	☒	A statement on whether measurements were taken from distinct samples or whether the same sample was measured repeatedly
☐	☒	The statistical test(s) used AND whether they are one- or two-sided<br>*Only common tests should be described solely by name; describe more complex techniques in the Methods section.*
☐	☒	A description of all covariates tested
☐	☒	A description of any assumptions or corrections, such as tests of normality and adjustment for multiple comparisons
☐	☒	A full description of the statistical parameters including central tendency (e.g. means) or other basic estimates (e.g. regression coefficient) AND variation (e.g. standard deviation) or associated estimates of uncertainty (e.g. confidence intervals)
☐	☒	For null hypothesis testing, the test statistic (e.g. *F*, *t*, *r*) with confidence intervals, effect sizes, degrees of freedom and *P* value noted<br>*Give P values as exact values whenever suitable.*
☒	☐	For Bayesian analysis, information on the choice of priors and Markov chain Monte Carlo settings
☒	☐	For hierarchical and complex designs, identification of the appropriate level for tests and full reporting of outcomes
☒	☐	Estimates of effect sizes (e.g. Cohen's *d*, Pearson's *r*), indicating how they were calculated

Our web collection on statistics for biologists contains articles on many of the points above.

Software and code

Policy information about availability of computer code

Data collection	no customized software was used
Data analysis	no customized software was used

For manuscripts utilizing custom algorithms or software that are central to the research but not yet described in published literature, software must be made available to editors/reviewers. We strongly encourage code deposition in a community repository (e.g. GitHub). See the Nature Research guidelines for submitting code & software for further information.

Data

Policy information about availability of data

All manuscripts must include a data availability statement. This statement should provide the following information, where applicable:
- Accession codes, unique identifiers, or web links for publicly available datasets
- A list of figures that have associated raw data
- A description of any restrictions on data availability

The authors declare that the data supporting the findings of this study are available within the paper and its supplementary information files.

Field-specific reporting

Please select the one below that is the best fit for your research. If you are not sure, read the appropriate sections before making your selection.

☒ Life sciences ☐ Behavioural & social sciences ☐ Ecological, evolutionary & environmental sciences

For a reference copy of the document with all sections, see nature.com/documents/nr-reporting-summary-flat.pdf

Life sciences study design

All studies must disclose on these points even when the disclosure is negative.

Sample size	Sample size was chosen to assure reproducibility of the experiments in accordance with the replacement, reduction and refinement principles of animal ethics regulation
Data exclusions	No data exclusions
Replication	Replicates were performed as described in the Methods and legends of the manuscript and the authors confirm that all attempts at replications were successful
Randomization	Randomization was not appropriate for this experimental approach
Blinding	Blinding was not performed in this study. A limited number of experimenters (M.G., JM.W and N.B.) are authorized to treat and perform tumor measurements in the SPF animal facility

Reporting for specific materials, systems and methods

We require information from authors about some types of materials, experimental systems and methods used in many studies. Here, indicate whether each material, system or method listed is relevant to your study. If you are not sure if a list item applies to your research, read the appropriate section before selecting a response.

Materials & experimental systems

n/a	Involved in the study	
☐	☒	Antibodies
☒	☐	Eukaryotic cell lines
☒	☐	Palaeontology
☐	☒	Animals and other organisms
☒	☐	Human research participants
☒	☐	Clinical data

Methods

n/a	Involved in the study	
☒	☐	ChIP-seq
☐	☒	Flow cytometry
☒	☐	MRI-based neuroimaging

Antibodies

Antibodies used	anti-IgG1 (#BE0083 : clone MOPC-21), IgG2a (#BE0089 : clone 2A3) , anti-TGFb (#BE0057 : clone 1D11.16.8), anti-IFNAR (#BE0241, clone Mar1-5A3), anti-CD8 (#BE0004-1 : clone 53-6.72), anti-GR1 (#BE0075 : clone RB6-8C5) , all purchased from Bioxcell.CD11b-BV421 (#562605), CD64-APC (#558539), CD11c-PeCy7 (#557401), TCRβ-BV605 (#562840) and CD4-BV711 (#563050), EpCAM-BV421 (#563214), CD31-Biot (#553371), streptavidin-PE (#554061) all purchased from BD pharmingen. CD45-AF700 (#103128), Ly6C-APCCy7 (#128025), Ly6G-BV510 (#127633), F4/80 BV650 (#123149), CD206-PE (#141706), Gp38-PE (#127408) all purchased from Biolegend. IA/IE-BV785 (#107645), CD8-PerCPef710 (#46-0081-82) from eBioscience. pIRF3-AF647 (#103275) and pTBK1-PE (#13498), p-SMAD2/3 (#8685) all purchased from Cell signaling. F4/80-AF488 (#MCA497A647) or AF647 (#MCA497A488) both purchased from Bio Rad. Goat anti-rabbit AF488 (#A11070) from life technologies.
Validation	All antibodies used in this study were validated on positive samples by flow cytometry or by immunofluorescence.

Animals and other organisms

Policy information about studies involving animals; ARRIVE guidelines recommended for reporting animal research

Laboratory animals	FvB/NCrl mice from Charles River laboratories
Wild animals	This study did not involve wild animals
Field-collected samples	This study did not involve field collected samples
Ethics oversight	All procedures used in this study were approved by the French animal experimentation and ethic committee of Paris Descartes University (CEEA 34, 16-063). Animal care was performed in compliance with all relevant ethical regulations for animal testing and research of the Federation of European Laboratory Animal Science association.

Note that full information on the approval of the study protocol must also be provided in the manuscript.

Flow Cytometry

Plots

Confirm that:

☒ The axis labels state the marker and fluorochrome used (e.g. CD4-FITC).

☒ The axis scales are clearly visible. Include numbers along axes only for bottom left plot of group (a 'group' is an analysis of identical markers).

☒ All plots are contour plots with outliers or pseudocolor plots.

☒ A numerical value for number of cells or percentage (with statistics) is provided.

Methodology

Sample preparation	Sample preparation were performed as described in the Methods.
Instrument	FORTESSA, LSR II and ARIA III
Software	FACS DIVA (Version 6 and 8) for sample collection and FlowJo (Version 10) for data analysis
Cell population abundance	50000 to 300000 TAM MHC II+ and MHCII- were sorted from pooled tumors per mouse. Purity was assessed with myeloid (F4/80, CD11b, ..) and MHC II cell markers
Gating strategy	Gating strategy is illustrated in the Supplementary Information

☒ Tick this box to confirm that a figure exemplifying the gating strategy is provided in the Supplementary Information.
